# Personality traits and psychological distress in Chinese adolescents: the mediating roles of anxiety and depression

**DOI:** 10.3389/fpsyg.2025.1748370

**Published:** 2026-01-16

**Authors:** Aijun Zhu, Di Xue, Huaijie Yang, Yanfang Ren

**Affiliations:** 1 The First College of Clinical Medical Science, China Three Gorges University, Yichang, China; 2 College of Medicine and Health Sciences, China Three Gorges University, Yichang, China; 3 Yichang Central People's Hospital, Yichang, China; 4 Yichang Special Care Hospital, Yichang, China

**Keywords:** adolescents, anxiety, chain mediating effects, depression, personality traits, psychological distress

## Abstract

**Background:**

Adolescents’ mental health is significantly influenced by their personality traits, particularly neuroticism, extraversion, and psychoticism. Understanding how these traits influence levels of psychological distress through anxiety and depression is crucial for developing effective interventions. This study aims to investigate the chain-mediated role of anxiety and depression in the relationship between adolescent personality traits and psychological distress.

**Methods:**

A cross-sectional study was conducted using convenience sampling among 3,673 adolescents. All participants completed self-report questionnaires including the Eysenck Personality Questionnaire (EPQ), Zung Self-Rating Anxiety Scale (SAS), Zung Self-Rating Depression Scale (SDS), and Symptom Checklist-90 (SCL-90). Structural equation modeling was used to test the direct and indirect relationships between personality traits, anxiety, depression, and psychological distress. The mediation model was tested using the bias-corrected percentile bootstrap method.

**Results:**

The results indicated that psychoticism exhibited a positive correlation with both adolescent anxiety and overall psychological distress. Neuroticism demonstrated a positive correlation with adolescent anxiety, depression, and overall psychological distress. Conversely, extraversion exhibited a negative correlation with adolescent anxiety, depression, and overall psychological distress. Anxiety was identified as a significant mediating variable between adolescent personality traits and overall psychological distress, whilst depression exerted a partial mediating effect within these relationships. Furthermore, anxiety and depression jointly produced a significant chain-mediated effect on the relationship between personality traits and psychological distress.

**Conclusion:**

This study reveals the underlying associative mechanisms through which personality traits influence overall psychological distress levels. These results underscore the value of integrating personality assessments with routine screening for anxiety and depression to better characterize adolescents’ risk profiles. This study contributes to informing practical guidelines for the prevention and intervention of adolescent mental health.

## 1 Introduction

Adolescence represents a period of heightened neurobiological and social sensitivity during which internalizing problems such as anxiety and depression frequently emerge and co-occur (Blakemore, 2019; Schmidt-Persson et al., 2024). A cross-border epidemiological surveillance data shows that emotional symptoms among teenagers have been rising significantly and persistently. Nearly one-third of American teenagers and approximately one-fourth of European teenagers have reported clinically significant poor mental health conditions (Schmidt-Persson et al., 2024). These trends hold significant clinical importance, as early internalizing symptoms frequently lead to persistent distress, academic difficulties, and an increased risk of subsequent mental illness. Although exogenous stress factors, such as being immersed in the digital world, are precursors to persistent emotional distress, functional disorders, and an increased risk of mental illness in adulthood (Witowska et al., 2025), personality differences that reflect an individual’s emotional, cognitive, and behavioral stability tendencies also crucial shape how adolescents cope with stress, regulate emotions, and participate in the social environment (Favini et al., 2025). According to Eysenck’s personality theory, personality is the product of an interaction between temperament (genetically determined nervous system types) and socialization experiences. Different personality traits will lead individuals to develop unique psychological adaptation patterns, thereby influencing mental health (Pan et al., 2024). Thus, personality traits may act as upstream determinants that predispose adolescents to anxiety and depression, which in turn contribute to broader mental health impairment. Currently, research on adolescent mental health is prevalent. However, participants in such studies predominantly originate from school settings (Burnell et al., 2025) or community contexts (Barker et al., 2025). Research on adolescent mental health within clinical adolescent samples remains relatively limited. Compared to their school and community counterparts, clinical adolescents exhibit lower levels of well-being (Lam et al., 2024), which in turn correlates with personality traits and mental health status (Rudolf, 2024). Consequently, exploring the personality traits and emotional states of clinical adolescent populations is crucial for medical and social development. This study will examine clinical adolescent populations to investigate the potential underlying mechanisms linking personality traits to psychological distress.

Personality-psychopathology links are now documented across the lifespan (Wilson and Olino, 2021). Research indicates that neuroticism is the most effective individual risk factor, showing a strong and specific association with anxiety and depression in different adolescent samples (Chen et al., 2023; Wang X. et al., 2025). Conversely, extraversion typically exhibits a protective effect, buffering against the onset of internalizing symptoms, while psychoticism may confer risk through impulsive or antagonistic interpersonal styles that erode social support (Chen et al., 2023; Pontillo et al., 2019). Beck’s cognitive theory posits that individual personality traits shape cognitive styles, whereby negative cognitions about the self, the world, and the future directly influence emotional symptoms (Persons et al., 2023). Emotion regulation theory posits that personality traits influence individuals’ selection and implementation of strategies such as cognitive reappraisal and expressive suppression. When cognitive distortions remain uncorrected, negative emotions persistently accumulate and worsen, perpetuating a vicious cycle (Liang et al., 2022). Personality vulnerability leads to cognitive distortions and failed emotional regulation, ultimately increasing psychological distress and jeopardizing mental health. Therefore, elucidating these associative mechanisms is crucial, as it clarifies the targets for preventive interventions, thereby mitigating the effects of underlying personality vulnerabilities.

The intrinsic mechanisms by which neuroticism influences clinical outcomes is the most extensively researched (Faur et al., 2025). Adolescents high in neuroticism display a well-documented profile of heightened threat sensitivity, attentional bias toward negative stimuli, and maladaptive emotion regulation strategies (e.g., rumination), which collectively predispose them to anxious hyperarousal and, subsequently, depressive anhedonia (Chen et al., 2023). This temporal sequence, wherein anxiety often precedes and potentiates depression, is supported by longitudinal studies (Marcos-Escobar et al., 2025). Research has found that anxiety symptoms can lead to subsequent worsening of depressive symptoms, with anhedonia—a core feature of depression—acting as a key mediating factor (Penninx et al., 2021). This pattern aligns with theoretical models positing that chronic anxiety consumes cognitive resources and diminishes engagement in rewarding activities, thereby fostering the emergence of depression (Marcos-Escobar et al., 2025). A sequential model tracing the progression from personality traits to anxiety, subsequently developing into depression, and ultimately influencing levels of psychological distress, provides a concise and testable framework for understanding the developmental process of internalizing comorbidities. Notably, these intrinsic connections possess plasticity. A recent cluster-randomized trial demonstrated that a brief, family-based intervention reducing leisure screen time significantly improved internalizing symptoms in youth, underscoring the malleability of the proposed mediating processes (Schmidt-Persson et al., 2024). This finding further substantiates the clinical significance of delineating the sequence of mediating links from stable personality traits to transient emotional states and on to broader psychological distress.

Although prior research has established associations between personality traits, anxiety, depression, and psychological well-being (Chervonsky and Hunt, 2019), the underlying mechanisms remain incompletely elucidated. Notably, existing studies predominantly treat anxiety and depression as parallel mediating variables (Landa-Blanco et al., 2024; Zhan et al., 2025), failing to account for their potential sequential and developmental relationships. The developmental cascade model posits (Fry and Hale, 2000) that an individual’s capabilities or difficulties within a particular developmental stage or domain (such as cognitive, emotional, or social) may propagate over time and influence development in other domains. This ultimately leads to long-term, significant, and often irreversible adaptive outcomes (Fry and Hale, 2000). Multiple childhood risks, such as family stress, may trigger tendencies towards anxiety or depression. These internalized issues continue to interact throughout development, ultimately influencing symptoms in early adulthood (Lee et al., 2024). Adolescence, as the pivotal transition phase between childhood and adulthood, represents a golden window for intervention and transformation. Therefore, this study aims to propose and test a chained mediation model using a large clinical convenience sample of adolescents: anxiety precedes depression, and both jointly mediate the influence of personality traits on adolescents’ overall psychological distress. This model reflects the developmental trajectory of internalizing symptoms and provides a more precise explanation of the mechanisms through which personality-related vulnerability evolves over time. Moreover, by simultaneously examining the three major dimensions of psychoticism, neuroticism and extraversion, this study overcomes the limitations of previous research that primarily focused on neuroticism. The inclusion of the psychoticism dimension in particular enables a comprehensive assessment of the personality-psychopathology, while also highlighting this dimension’s unique and under-explored role in adolescent mental health.

**Figure 1:**
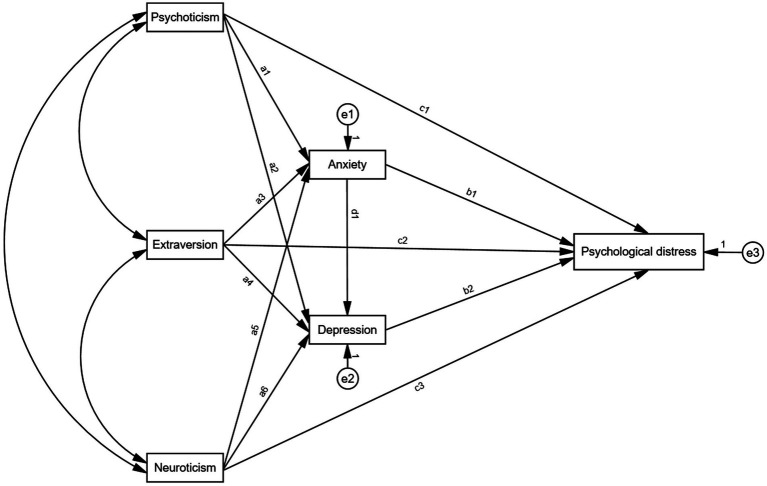
Hypothetical chain Mediation Model.

We established a theoretical hypothesis model (Figure 1) and put forward the following hypotheses:(1) Psychoticism is significantly positively correlated with adolescents’ anxiety, depression, and overall psychological distress. (2) Neuroticism is significantly positively correlated with adolescents’ anxiety, depression, and overall psychological distress. (3) Extraversion is significantly negatively correlated with adolescents’ anxiety, depression, and overall psychological distress. (4) Anxiety plays a mediating role in the relationship between personality traits and psychological distress. (5) Depression plays a mediating role in the relationship between personality traits and psychological distress. (6) Anxiety and depression have a chain-mediated effect between adolescents’ personality traits and levels of psychological distress.

## 2 Methods

### 2.1 Participants

A cross-sectional study was conducted using convenience sampling among 3,673 adolescents. All participants were from teenagers who visited the psychological outpatient department of a certain hospital in Yichang City, Hubei Province. The entire recruitment process lasted from August 2023 to March 2025. All participants completed the Eysenck Personality Questionnaire (EPQ), the Self-Rating Anxiety Scale (SAS), the Self-Rating Depression Scale (SDS), and the Symptom Checklist 90 (SCL-90). Each participant signed an informed consent form before officially participating. Participants were selected based on the following inclusion criteria: (1) Age≤18 years. (2) Clear consciousness with no cognitive development defects. (3) Willingness to participate in questionnaire surveys. Exclusion criteria included: (1) Mental retardation. (2) Recent psychiatric medication use. (3) Inability or refusal to participate in the survey. (4) Participants who selected the same response option for all questionnaire items. The study was approved by the Medical Ethics Review Committee of Yichang Special Care Hospital.

A total of 3,750 questionnaires were collected in this study. After rigorous screening, 77 questionnaires with missing values were excluded, resulting in 3673 valid questionnaires, with an effective response rate of 97.9%. The demographic characteristics of the sample showed that there were 1,255 males (34.2%) and 2,418 females (65.8%). The average age of the participants was 15.14 years (SD = 1.72), with the age range spanning from 9 to 18 years.

### 2.2 Measures

#### 2.2.1 Personality

The Eysenck Personality Questionnaire (EPQ) was utilized to assess core personality traits. This instrument is designed to evaluate how individuals react to stress and their emotional stability, alongside sociability and positivity traits (Chen X. et al., 2021). In our study, the adolescent questionnaire by Gong containing 88 questions (Gong, 1984) was applied to investigate personality characteristics of adolescents. The EPQ consists of 88 items, divided into four dimensions: neuroticism, extraversion, psychoticism, and a lie scale to ensure response validity. Participants answered each question using a binary (yes/no) response, with higher scores indicating greater psychoticism, neuroticism or extraversion. The EPQ has been widely applied in the Chinese context due to its strong psychometric properties and straightforward approach to personality assessment (Chen X. et al., 2021).

#### 2.2.2 Psychological distress

The Symptom Checklist-90 (SCL-90) consists of 90 individual items and covers a highly comprehensive range of psychiatric symptoms. These symptoms encompass diverse aspects such as subjective feelings, emotional responses, thought processes, consciousness states, behavioral traits, lifestyle habits, interpersonal relationships, dietary intake, and sleep quality (Wei et al., 2018). It is primarily used to assess the range of psychological symptoms exhibited by subjects and their severity. The SCL-90 demonstrates strong psychometric characteristics and cross-cultural applicability in the Chinese context (Zhou et al., 2021). In this study, participants rated the severity of their symptoms over the past week on a 5-point Likert scale, ranging from 1 (not at all) to 5 (extremely). Higher scores indicate greater psychological distress.

#### 2.2.3 Anxiety

The Self-Rating Anxiety Scale (SAS) was used to assess the level of anxiety experienced by participants. The SAS is a self-report tool designed to measure the frequency and severity of anxiety symptoms, focusing on physical and cognitive manifestations of anxiety (Zung, 1971). The SAS has been widely used in both clinical and research settings, and its reliability and validity have been confirmed across diverse populations (Liu, 2025; Pang et al., 2019; Ruan et al., 2024). The scale consists of 20 items, each rated on a 4-point Likert scale, ranging from 1 (none or little of the time) to 4 (most or all of the time). Higher scores indicate higher levels of anxiety.

#### 2.2.4 Depression

The Self-Rating Depression Scale (SDS) was used to measure depressive symptoms in the participants. The SDS is a widely used self-report tool that assesses the severity of depression by evaluating mood, behavior, and physical symptoms related to depression (Zung, 1965). The scale includes 20 items that reflect various aspects of depressive symptoms, such as sadness, loss of interest, and fatigue. Each item is rated on a 4-point Likert scale, ranging from 1 (a little of the time) to 4 (most or all of the time). Higher scores indicate more severe depressive symptoms. The Self-Rating Depression Scale (SDS) has been validated in the Chinese context and is regarded as a reliable and effective tool for assessing depression (Ruan et al., 2024).

### 2.3 Data analysis

Data analysis was performed using SPSS 27.0 for descriptive statistics. For categorical variables, the frequency (n) and percentage (%) were calculated. Continuous variables were assessed for normality using the Kolmogorov–Smirnov test, and data were presented as mean ± standard deviation for normally distributed variables or median (M) and interquartile range (P25, P75) for skewed variables. To assess the relationships between the variables, Pearson correlation coefficients were computed. Mediation effects were tested using structural equation modeling (SEM) conducted via AMOS 29.0, with bias-corrected bootstrap sampling (5,000 repetitions) used to test the significance of indirect effects, and the model fit was evaluated using χ2/df, comparative fit index (CFI), Tucker–Lewis index (TLI), root mean square error of approximation (RMSEA), and SRMR indicators. If χ2/df < 5, CFI > 0.9, TLI > 0.9, RMSEA<0.08, and SRMR<0.08, the model was considered to fit the data well (Chen W. et al., 2021). The significance level was set at *α* = 0.05.

## 3 Results

### 3.1 Descriptive statistics and correlation analysis of variables

As shown in Table 1, correlations among personality traits, anxiety, depression, and overall psychological distress were consistently significant (*p* < 0.01). The closest linkage was between anxiety and depression (*r* = 0.846, *p* < 0.01), and both were strongly aligned with poorer overall psychological distress (anxiety: *r* = 0.858, *p* < 0.01; depression: *r* = 0.816, *p* < 0.01). Among traits, neuroticism showed the most pronounced associations with anxiety and depression symptoms (depression: *r* = 0.711, *p* < 0.01; anxiety: *r* = 0.663, *p* < 0.01; psychological distress: *r* = 0.696, *p* < 0.01), indicating that higher emotional instability co-occurs with markedly elevated psychopathology. Extraversion demonstrated protective inverse relations (depression: *r* = −0.333, *p* < 0.01; anxiety: *r* = −0.233, *p* < 0.01; psychological distress: *r* = −0.265, *p* < 0.01). Psychoticism correlated positively but more modestly with anxiety and depression symptoms (depression: *r* = 0.282, *p* < 0.01; anxiety: *r* = 0.290, *p* < 0.01; psychological distress: *r* = 0.318, *p* < 0.01). Trait interrelations were in expected directions (e.g., extraversion-neuroticism *r* = −0.156, *p* < 0.01), together supporting a framework that personality, especially neuroticism, is mainly associated with overall psychological distress through anxiety and depression.

**Table 1:** Scores and correlations of EPQ, SAS, SDS and SCL-90 scales [*n* = 3,673 points, M (P25, P75)].

Variable	Median (25–75 percentile)	Psychoticism	Extraversion	Neuroticism	Depression	Anxiety	Psychological distress
Psychoticism	50(40–60)	1.000					
Extraversion	50(40–60)	−0.249**	1.000				
Neuroticism	70(65–75)	0.179**	−0.156**	1.000			
Depression	73(63–80)	0.282**	−0.333**	0.711^**^	1.000		
Anxiety	60(49–69)	0.290**	−0.233**	0.663^**^	0.846^**^	1.000	
Psychological distress	261(199–313)	0.318**	−0.265**	0.696^**^	0.816^**^	0.858^**^	1.000

“**” means *p* < 0.01.

### 3.2 Chain mediation model analysis

**Figure 2:**
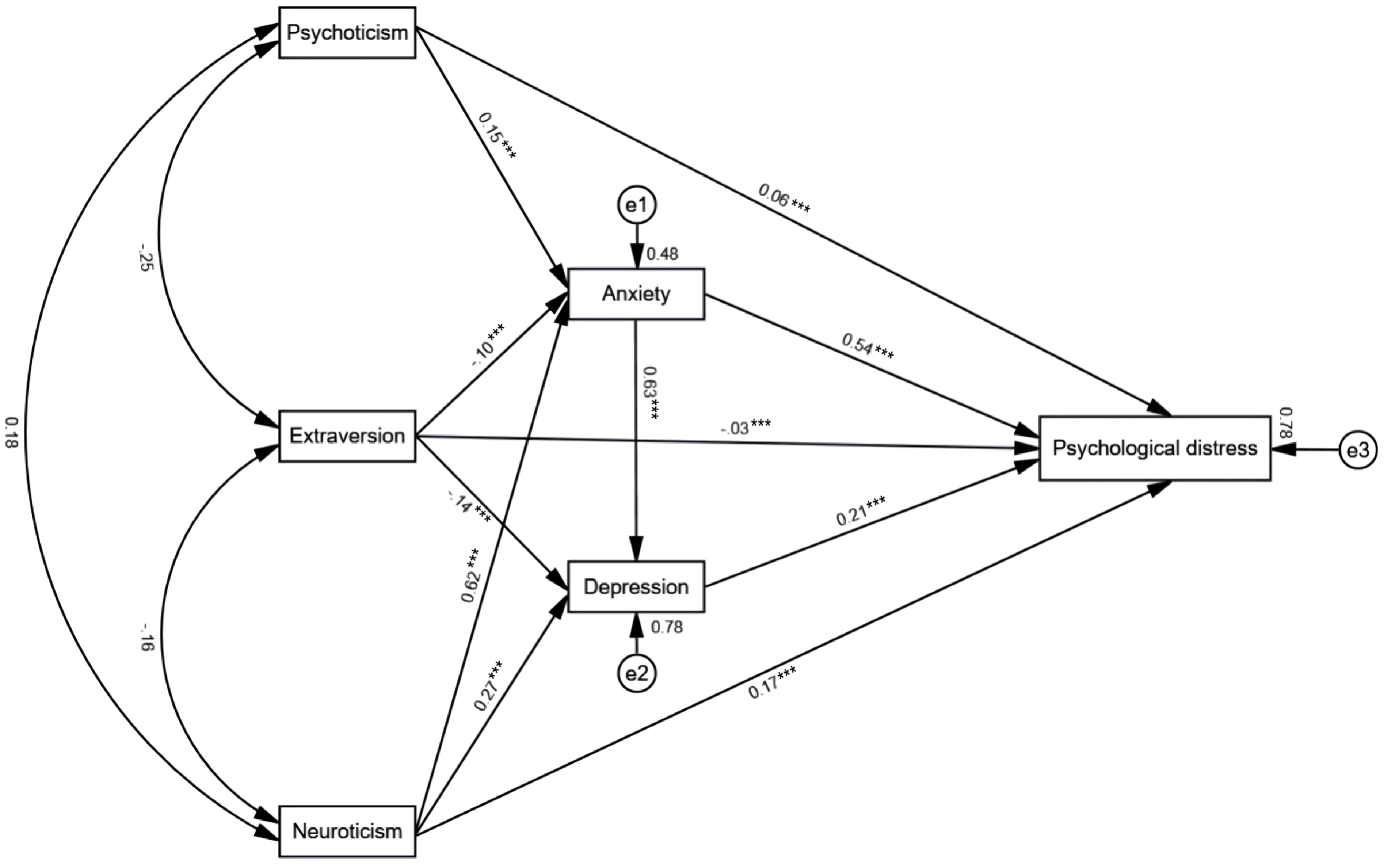
Chain mediation model. ***means *p* < 0.001.

The mediating model analysis was constructed using Amos 29 software. The analysis results of the mediation model showed that the path coefficients from Psychoticism to depressive symptoms were not significant (*β* = 0.02, *p* = 0.056). Therefore, the non-significant paths were removed, and then the mediation model was reanalyze. The results of the mediating effect between personality traits and mental health are shown in Figure 2. The fit indices for the modified model were acceptable: χ^2^/df = 3.585, CFI = 0.999, TLI = 0.997, SRMR = 0.0031, RMSEA (90%CI) = 0.027(0.000–0.059) (Figure 2). As shown in Figure 2, the path analysis in the model indicated that adolescent psychoticism had a significant positive predictive effect on anxiety (*β* = 0.15, *p* < 0.001) and psychological distress (*β* = 0.06, *p* < 0.001); adolescent neuroticism had a significant positive predictive effect on anxiety (*β* = 0.62, *p* < 0.001), depression (*β* = 0.27, *p* < 0.001), and psychological distress (*β* = 0.17, *p* < 0.001); adolescent extraversion had a significant negative predictive effect on anxiety (*β* = −0.10, *p* < 0.001), depression (*β* = −0.14, *p* < 0.001), and psychological distress (*β* = −0.03, *p* < 0.001); adolescent anxiety had a significant positive predictive effect on depression (*β* = 0.63, *p* < 0.001) and psychological distress (*β* = 0.54, *p* < 0.001); adolescent depression had a significant positive predictive effect on psychological distress (*β* = 0.21, *p* < 0.001).

### 3.3 Chain mediation effect test

The chain mediation test showed that all proposed indirect associations between personality traits and overall psychological distress via anxiety and/or depression were statistically significant when evaluated with bias-corrected bootstrapping, with 95% confidence intervals that did not include zero, confirming the presence of robust mediation effects (Table 2). Across traits, anxiety emerged as the principal conduit, and the sequential anxiety → depression linkage contributed additional unique mediation. For psychoticism, the total effect on psychological distress was positive (total effect = 0.169, 95% CI = [0.146, 0.191], *p* < 0.001), with nearly two-thirds transmitted indirectly (total indirect = 0.105, 95% CI = [0.088, 0.121], *p* < 0.001). The dominant path was psychoticism → anxiety → psychological distress (Ind1 = 0.085, SE = 0.007, 95% CI = [0.071, 0.099], *p* < 0.001), indicating that higher psychoticism elevates anxiety, which in turn increase overall psychological distress. The sequential chain psychoticism → anxiety → depression → psychological distress was also significant though smaller (Ind2 = 0.020, SE = 0.002, 95% CI = [0.016, 0.025], *p* < 0.001). A residual direct association remained (direct effect = 0.064, SE = 0.008, 95% CI = [0.049, 0.080], *p* < 0.001), supporting partial rather than full mediation. Together, these effects delineate a mechanism in which psychoticism primarily impacts psychological distress indirectly through anxiety, with a smaller additive chain via depression, and a statistically reliable direct component (Table 2).

**Table 2:** Chain mediating effect test.

Effect	Path correlation	Effect value	Boot SE	Boot LLCI	Boot ULCI	Effect size
Indirect effect						
Ind1	Psychoticism → anxiety → psychological distress	0.085	0.007	0.071	0.099	50.3%
Ind2	Psychoticism → anxiety → depression → psychological distress	0.020	0.002	0.016	0.025	11.8%
Total indirect effect	Ind1–Ind2	0.105	0.009	0.088	0.121	62.1%
Direct effect	Psychoticism → psychological distress	0.064	0.008	0.049	0.080	37.9%
Total effect	Direct effect–indirect effect	0.169	0.011	0.146	0.191	100%
Indirect effect						
Ind3	Extraversion → anxiety → psychological distress	−0.053	0.007	−0.066	−0.040	43.4%
Ind4	Extraversion → depression → psychological distress	−0.029	0.003	−0.035	−0.024	23.8%
Ind5	Extraversion → anxiety → depression → psychological distress	−0.013	0.002	−0.017	−0.009	10.7%
Total indirect effect	Ind3–Ind4–Ind5	−0.095	0.009	−0.112	−0.077	77.9%
Direct effect	Extraversion → psychological distress	−0.027	0.008	−0.044	−0.011	22.1%
Total effect	Direct effect–indirect effect	−0.122	0.012	−0.145	−0.100	100%
Indirect effect						
Ind6	Neuroticism → anxiety → psychological distress	0.338	0.010	0.318	0.359	52.3%
Ind7	Neuroticism → depression → psychological distress	0.055	0.005	0.045	0.065	8.5%
Ind8	Neuroticism → anxiety → depression → psychological distress	0.081	0.007	0.068	0.093	12.5%
Total indirect effect	Ind6–Ind7–Ind8	0.474	0.010	0.455	0.493	73.3%
Direct effect	Neuroticism → psychological distress	0.173	0.011	0.151	0.195	26.7%
Total effect	Direct effect–Indirect effect	0.647	0.009	0.629	0.665	100%

For extraversion, the effects were uniformly protective and again largely mediated. The total effect was negative (total effect = −0.122, 95% CI = [−0.145, −0.100], *p* < 0.001), with the bulk conveyed by the mediators (total indirect = −0.095, 95% CI = [−0.112, −0.077], *p* < 0.001). The strongest single route was extraversion → anxiety → psychological distress (Ind3 = −0.053, SE = 0.007, 95% CI = [−0.066, −0.040], p < 0.001), complemented by extraversion → depression → psychological distress (Ind4 = −0.029, SE = 0.003, 95% CI = [−0.035, −0.024], *p* < 0.001) and a significant chain extraversion → anxiety → depression → psychological distress (Ind5 = −0.013, SE = 0.002, 95% CI = [−0.017, −0.009], *p* < 0.001). A small, independent direct path persisted (direct effect = −0.027, SE = 0.008, 95% CI = [−0.044, −0.011], *p* = 0.001). Neuroticism displayed the largest total association with psychological distress (total effect = 0.647, 95% CI = [0.629, 0.665], *p* < 0.00), and mediation predominated (total indirect = 0.474, 95% CI = [0.455, 0.493], p < 0.001). The principal conduit was neuroticism → anxiety → psychological distress (Ind6 = 0.338, SE = 0.010, 95% CI = [0.318, 0.359], *p* < 0.001), supplemented by neuroticism → anxiety → depression → psychological distress (Ind8 = 0.081, SE = 0.007, 95% CI = [0.068, 0.093], *p* < 0.001) and neuroticism → depression → psychological distress (Ind7 = 0.055, SE = 0.005, 95% CI = [0.045, 0.065], *p* < 0.001). Even after accounting for these mediated routes, neuroticism retained a sizable direct effect (direct effect = 0.173, SE = 0.011, 95% CI = [0.151, 0.195], *p* < 0.001) (Table 2). These results indicate that anxiety and depression not only partially mediate the relationship between personality and overall psychological distress, but also have a chain mediating effect on them.

## 4 Discussion

This study validated a coherent and statistically significant chain mediation model, revealing the associative structure among personality traits, emotional symptoms, and psychological distress. The research first examined the relationship between adolescent personality traits and mental health status, finding that personality traits have a significant positive or negative predictive effect on psychological distress (Kang et al., 2023; Pan et al., 2024). Correlation analysis revealed that higher levels of neuroticism and psychoticism were significantly associated with more severe anxiety and depressive symptoms, as well as increased overall psychological distress. Conversely, extraversion exhibited a protective association, showing a negative correlation with these outcomes. Adolescence is a period of significant physical, psychological and cognitive transformation. Strengthening identity formation during this stage enables young people to develop a clearer and more resolute sense of self, thereby enhancing their capacity to perceive life’s meaning and improve mental wellbeing (Ruiz and Yabut, 2024). Consequently, personality assessment should be integrated into mental health risk evaluations, with neuroticism and psychoticism serving as indicators of emotional dysregulation susceptibility, while extraversion—acting as a buffering factor—should be applied to identify high-risk individuals. Whilst respecting adolescents’ quest for independent identity, we must dedicate greater effort to assisting them in exploring and establishing a clear, positive self-identity, thereby fostering a well-rounded personality. When adolescents possess such a well-rounded personality, they are more likely to contemplate and explore life’s meaning, and are also more likely to develop healthy psychological functioning (Yang et al., 2025).

This study found that anxiety fully mediates the relationship between adolescent personality traits and psychological distress, whereas depression partially mediates the association between adolescent personality traits and psychological distress. This indicates that anxiety occupies a relatively more central position between personality traits and psychological distress, while depression further exacerbates psychological impairment on top of anxiety. This implies that the more intense adolescents’ anxiety responses are, the more likely they are to become trapped in a vicious cycle of negative emotions, leading to social problems and social isolation from peers, ultimately triggering various psychological disorders (Fu et al., 2025). Notably, compared to studies emphasizing self-efficacy or behavioral mediating variables (Cai et al., 2024), this research model establishes emotional processes as the core mediator between personality and overall psychological distress, highlighting the pivotal role of anxiety regulation in adolescent development. This distinction may reflect developmental specificity: adolescents typically exhibit limited metacognitive control and heightened emotional reactivity, rendering anxiety a pivotal entry point for psychopathology (Chen et al., 2023). Concurrently, the relative stability of personality traits during adolescence suggests these emotional mediating factors are more amenable to change than the traits themselves (Bleidorn et al., 2022). Therefore, early intervention measures should focus on anxiety management as a crucial entry point for clinical screening and early intervention. Strategies such as cognitive behavioural therapy or mindfulness-based therapies should be employed to interrupt the progression of excessive anxiety towards depressive withdrawal. By strengthening social interaction and emotional regulation training, one can both harness the buffering effect of extroverted personality traits and counteract the rigid tendencies stemming from neurotic dispositions.

The study found that anxiety and depression played a chained mediating role between personality traits and psychological distress. Personality traits (particularly neuroticism) were closely associated with heightened anxiety, which in turn was linked to depressive symptoms. Together, these two factors accounted for the vast majority of the influence of personality traits on psychological distress. These findings support the hypothesis that personality traits primarily link to adolescent psychological distress through internalized emotional processes. This suggests anxiety serves as the proximal emotional response of trait susceptibility, while depression represents the downstream consequence of sustained distress. This associational pattern aligns with previously proposed chain reaction models and supports developmental models of internalizing comorbidity (Collins et al., 2025). Simultaneously, these findings transcend common models treating anxiety and depression as parallel mediators, offering a progressive perspective on adolescent internalizing problems consistent with developmental cascade models. Here, stable vulnerability traits trigger sequential emotional states, ultimately leading to broader, more enduring psychological maladaptation. Within this framework, anxiety functions as an ‘emotional amplifier’ and proximal mechanism for personality traits, while depression serves as a secondary yet critical emotional mechanism that amplifies anxiety’s impact. The significant positive predictive effect of anxiety on depression (*β* = 0.63) in this study exemplifies this process. For individuals exhibiting heightened neuroticism (Chen et al., 2023) or psychoticism (Mullins-Sweatt et al., 2019), anxiety immediately amplifies emotional intensity, increasing the likelihood of adopting negative coping strategies and social withdrawal. Depression then emerges as the ‘endpoint’ of this emotional trajectory, reflecting a state of chronic stress internalization and the breakdown of emotional regulation systems (Barlow et al., 2014). Conversely, extraversion buffers the transition from anxiety to depression by promoting positive emotions, social support, and flexible coping (Stein et al., 2021). This linkage between discrete personality traits and cross-diagnostic emotional dysregulation processes underscores the potential value of interventions targeting shared emotional regulation mechanisms. Research indicates that negative emotions such as depression, anxiety and stress are prevalent and serious issues among adolescents, impacting their psychological and physical health, academic performance and quality of life (Geng et al., 2020). The origins of negative emotions among adolescents are diverse and complex, encompassing academic pressure, competition, and social pressures (Wang J. et al., 2025). Therefore, we should prioritize developing adolescents’ emotional regulation capabilities rather than attempting to alter personality traits themselves. Targeting emotional responses such as anxiety and depression as intervention objectives, strategies should integrate emotional regulation training, school-based mindfulness programs, and measures to enhance parent–child communication. These approaches can mitigate the anxiety-depression cascade effect. By intervening early in emotional dysregulation and its environmental reinforcing factors, these approaches can interrupt the progression from anxiety to depression, thereby shielding adolescents’ mental wellbeing from the cumulative effects of maladaptive personality expression.

In general, our research not only reveals a potential chain-reaction-based associative mechanism linking adolescent personality traits to their overall psychological distress, highlighting the pivotal role of internal emotional processes—particularly anxiety and depression—within this relationship, but also holds practical significance for guiding adolescent education policy, intervention development, and school management practices. This is especially pertinent within today’s rapidly evolving, highly competitive social environment.

## 5 Limitations

The limitations of this study are as follows: (1) This is a cross-sectional survey and cannot examine the long-term changes and causal relationships between personality and psychological distress. Owing to the inability to track changes in the variables over time, the research results can only reflect the current state. Future studies should use longitudinal designs to reveal the causal relationships and trends between these variables at different time points. (2) The reliance on self-reported measures may introduce response biases, and incorporating objective assessments could enhance the validity of the findings. The sample was drawn from a specific region, which may limit the generalizability of the results to broader populations. Future research should aim to include diverse samples to improve the external validity of the findings. (3) This study did not fully account for other factors that may influence psychological distress, such as adverse childhood experiences and social support. Therefore, future research should comprehensively consider these factors to better understand the mechanisms through which personality traits affect psychological distress. (4) This study focuses on an outpatient convenience sample, whose participants may inherently possess higher levels of help-seeking motivation and psychological distress. While this facilitates in-depth examination of clinically relevant mediating mechanisms, it also limits the direct generalizability of findings to broader, non-help-seeking adolescent populations. (5) The psychometric properties of the Psychoticism (P) scale within the Eysenck Personality Inventory may account for its weaker effect size compared to Neuroticism and Extraversion. Future research employing the Big Five personality model for replication studies could help elucidate the specific influences of these traits.

## 6 Conclusion

In conclusion, this study provides a comprehensive framework for understanding how adolescent personality traits relate to psychological distress through the sequential influence of anxiety and depression. The findings underscore the complex interplay between stable personality dispositions and dynamic emotional processes, suggesting that early emotional dysregulation serves as a pivotal mechanism in the development of broader psychological problems. Beyond highlighting the psychological mechanism involved, this work emphasizes the importance of integrating personality-based assessment into preventive psychological distress strategies during adolescence. Interventions that strengthen emotional regulation, resilience, and adaptive coping may help attenuate the maladaptive expression of high neuroticism or psychoticism and enhance the protective qualities of extraversion. Future research should employ longitudinal and cross-cultural designs to establish causal directions and explore potential biological or social moderators—such as parental behavior, peer climate, and digital media exposure—that shape the personality–emotion–psychological distress nexus. Moreover, multimethod approaches combining self-report, behavioral, and neurophysiological measures could provide a more nuanced understanding of how trait vulnerabilities translate into emotional dysfunction over time. Ultimately, advancing research in this domain will contribute to the early identification of high-risk adolescents and inform more precise, personality-tailored prevention and intervention programs aimed at promoting lifelong mental well-being.

## Data Availability

The original contributions presented in the study are included in the article/supplementary material, further inquiries can be directed to the corresponding author.
